# Dairy nutrition educational messages help increase dairy product knowledge, purchasing, and consumption

**DOI:** 10.3168/jdsc.2023-0417

**Published:** 2023-10-06

**Authors:** Jack Myers, Derek Schweiger, Stephanie Clark

**Affiliations:** 1 Department of Agricultural Economics and Agribusiness, University of Arkansas, Fayetteville, AR 72701; 2 Department of Food Science and Human Nutrition, Iowa State University, Ames, IA 50011

## Abstract

The objectives of this research were to educate those who consume an inadequate amount of dairy (less than 3 servings of dairy per day) about various topics related to dairy nutrition, test retention of information, and motivate purchasing and consumption of dairy products. Twenty-four nominal focus group (NFG) sessions were conducted (Ames, IA: April 2021, 10, n = 94; Manhattan, KS: 6 in July 2021 and 8 in October 2021, n = 101). The NFG were randomly assigned into control or treatment groups. The control only received a nutrition facts panel (NFP) label lesson. Treatment groups received the NFP label lesson, plus one of 3 different educational messages (EM; lactose maldigestion, 9 essential nutrients, and prebiotics and probiotics). Identical scripts were followed, except for the EM and infographics. Participants completed surveys at the beginning (pre-survey) and end (post-survey) of the NFG, and 1 mo later (follow-up). A total of 195 participants completed the NFG and pre- and post-surveys; 185 of those completed the follow-up survey. Nonparametric Wilcoxon signed-rank tests were run to determine if individuals learned and retained information and if they changed purchasing or consumption. Participants learned about dairy nutrition topics (validated by correct responses in survey questions) and increased their consumption and purchasing of cheese, ice cream, milk, and yogurt by at least 20% after participating in an NFG. Consumption did not increase enough for those who consume an inadequate amount of dairy to meet the recommended 21 servings of dairy per week. Adjustments to the EM and infographics may improve learning, knowledge retention, and more meaningfully increase dairy purchasing and consumption.

## Highlights

• Dairy nutrition infographics and scripted lessons were delivered to those who consume an inadequate amount of dairy.
• Dairy nutrition knowledge, purchasing, and consumption increased.
• Dairy nutrition messaging may motivate behavior change.

## Graphical Abstract

**Figure:**
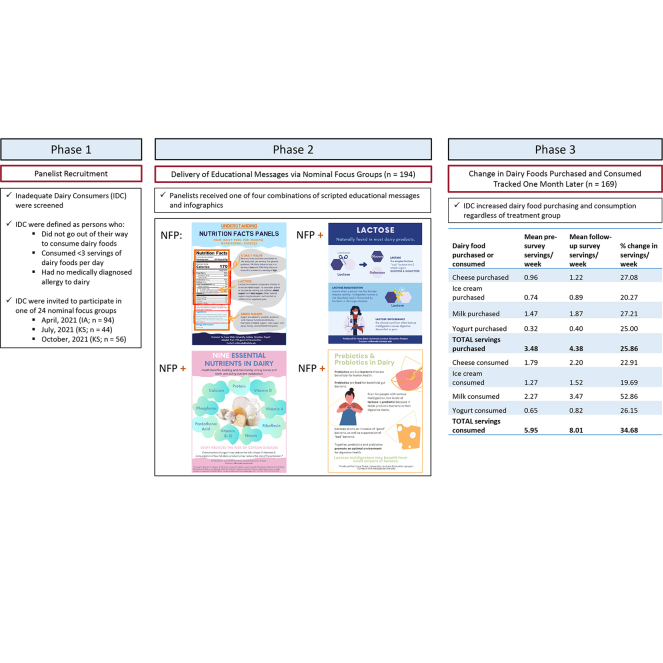
**Summary:** In recent years, dairy industry stakeholder groups have developed educational messages to try to increase dairy food purchasing and consumption among those who consume an inadequate amount of dairy in the United States. Four educational messages were developed and delivered to those who consume fewer than 3 servings of dairy per day, in nominal focus groups. Dairy purchasing and consumption by panelists was quantified before and after delivery of the educational messages to measure change resulting from the intervention. Panelists' purchasing and consumption of cheese, ice cream, milk, and yogurt increased by more than 20%.

The Dietary Guidelines for Americans, 2020–2025, recommends that a healthy adult who consumes 2,000 calories should include 3 cups or 3 cups equivalent of dairy or soy alternatives per day; however, males and females aged 19 to 59 yr, on average, are consuming about 2 and <1.5 cups of dairy a day, respectively (USDA and USDHHS, 2020). Inadequate dairy consumption has negative nutritional consequences, not only because of the loss of milk's essential nutrients (e.g., high quality protein, potassium, vitamin B_12_), but because lactose is a natural “prebiotic” (i.e., it nourishes lactic acid bacteria that may be considered “probiotic,” or beneficial, to human health; Markowiak and Śliżewska, 2017). Additionally, since reducing dairy product consumption reduces the intake of lactose, dairy avoidance can lead to the suppression of the lactase gene, the gene responsible for producing the enzyme (lactase-phlorizin hydrolase) that hydrolyzes lactose into glucose and galactose (Ugidos-Rodríguez et al., 2018). Dairy avoidance resulting from misinformation becomes intergenerational when parents with self-diagnosed lactose intolerance place their children on lactose-restricted diets (even in the absence of symptoms) in the mistaken belief that they will develop symptoms if given lactose (Suchy et al., 2010).

To combat the decline in milk consumption and to increase the consumption of dairy foods, groups such as Dairy Management Inc. have developed educational materials in a variety of formats (e.g., infographics, advertisements) and delivery platforms (e.g., social media influencers, print). In the present work, dairy nutrition educational messages (**EM**) and infographics were presented to inadequate dairy consumers (**IDC**), those who consume less than 3 servings of dairy per day, in nominal focus groups (**NFG**). A NFG is a structured research technique that limits interactions among panelists (de Ruyter, 1996). We proposed that this style of intervention could help increase dairy knowledge, purchasing, and consumption among IDC.

The study and related materials were approved for the involvement of human subjects by the Iowa State University Institutional Review Board, which carried through via a sub-award to Kansas State University. The use of 2 different geographic locations provided a wider net of panelists for the project. The study included 3 phases: (1) screening survey, (2) NFG, and (3) a follow-up survey. The project was completed at both locations in a very similar manner. Survey questions and response choices, human subject approvals (Institutional Review Board), NFG scripts, and infographics, as well as the ice cream samples (Schweiger et al., 2023), were the same. Only the participants' geographic locations and NFG schedules differed: Ames, Iowa, area (spring semester) and Manhattan, Kansas, area (summer and fall semesters). To maintain the structure of NFG, panelists could ask questions to the moderators but discussion was not encouraged among panelists. Four infographics were developed to help educate research participants about food labels and dairy concepts: nutrition facts panels (**NFP**), lactose maldigestion, 9 essential nutrients, and prebiotics and probiotics.

An invitation to participate in a screening survey was created for dissemination via listservs at both institutions. The food choices and intolerances screening survey was developed and delivered via Qualtrics (Provo, UT), and was accessible by anonymous links to the survey. The criteria for participants included being at least 18 yr of age and personally purchasing food and beverages at least once per month. The screening surveys were launched in Iowa in January, and in Kansas in May and August. This 15-question survey asked about proximity to Ames, Iowa, or Manhattan, Kansas (1 question), food purchasing and consumption (8 questions), NFP knowledge (4), discomfort after consuming foods (1), and medically diagnosed allergies or intolerances to foods (1). Questions solely related to dairy were not asked, as the researchers did not want to prime participants that this research was related to the dairy industry. However, dairy was one of the options in several questions, which were later used for screening. To compensate participants for their time to complete the screening survey, 2 randomly selected individuals received a $25 Amazon gift card in March, June, and September.

After the survey was closed at each time period, a funneling process was conducted. Potential NFG panelists were identified as those who (1) indicated interest to participate in a follow-up study with ice cream, (2) completed the screening survey in ≥80 s, (3) resided in the greater Ames (IA panelists) or Manhattan (KS panelists) areas, (4) did not go out of their way to consume dairy foods (because IDC were sought), (5) did not have a medically diagnosed allergy or intolerance to dairy foods (because the follow-up study involved consuming ice cream), and (6) were IDC (selected the option indicating that they consumed <3 servings of dairy foods per day). Although we did not invite those who indicated a medically diagnosed issue with dairy, people who avoided dairy were not removed from the NFG as they were given a clear indication that ice cream would be served at the NFG. It was believed that if they completely avoided dairy, they would not volunteer to participate. Additionally, if they attended the NFG, there was a chance we could educate them about the importance of consuming even a little dairy in their diet. Again, a goal of the research was to investigate the impact of a variety of messages on the potential to increase dairy purchasing and consumption.

Once IDC were identified, they were provided multiple dates and times to attend an NFG. Registrants were informed of the specific day, time, and location of the NFG, and asked to bring a cellular device for surveys.

Ten NFG were conducted in Ames, Iowa, in April 2021; 14 were conducted in Manhattan, Kansas (6 in July and 8 occurred in October 2021). Upon arrival, panelists were handed folders with randomly generated 3-digit codes and asked to sit at sanitized, physically separated tables that contained a napkin, spoon, 0.5 L (16.9 fl. oz.) bottle of water, sanitized pen, and cuspidor. Everything about the sessions was designed to be the same (facilitators followed a script) except the educational message (**EM**), which randomly differed among NFG. All panelists were seated facing the facilitators (at least 2 per session), who conducted each NFG according to a script (developed in preliminary work). Unlike traditional single-style focus groups, where data are collected from interacting panelists, the goal was to efficiently deliver educational information to the participants. Thus, interactions between the panelists were limited, but panelists were encouraged to ask questions to the moderators during the NFG.

After introductions of facilitators, one facilitator read the consent form in its entirety (running time approximately 7 min). Panelists could excuse themselves or sign the consent form to proceed. Next, a 34-question pre-survey was administered via Qualtrics using a QR code (running time approximately 17–20 min). Questions focused on demographics (4 questions), general food consumption (3), nutrition and dairy knowledge (10), and dairy purchasing and consumption behaviors (16).

Next, facilitators walked the participants through the NFP, ingredients, and allergen statement lesson (running time approximately 23–25 min). Panelists were given an opportunity to ask questions at 2 points during and following the NFP lesson. Percent daily value (**%DV**) was explained in the context of ice cream. Facilitators explained that lactose is not an added sugar because it is naturally present in milk and were shown how to determine the amount of lactose present in dairy products based upon information provided in the NFP. Panelists were encouraged to take the infographic with them. Those who *only* received this lesson were part of the control group.

An ice cream acceptability test (detailed in Schweiger et al., 2023) was conducted next (running time approximately 32–37 min). In brief, three 3-digit-coded samples of ice cream were served in randomized order. After tasting, NFP and ingredient statements were explained, with particular attention to differences in lactose and added sugar. Panelists were given an opportunity to ask questions (running time approximately 35–38 min).

At this point, according to the randomization scheme, 3 out of 4 of the NFG received an additional EM (approximately 3 min added to running time). One out of 4 NFG did not receive an additional EM (no additional running time). One infographic, specific to each additional EM, was provided to aid in understanding the material read to them during their session. Panelists were encouraged to ask questions and to take infographics with them. The lactose maldigestion EM was designed to explain that lactose is a disaccharide (glucose and galactose) that must be broken into those sugars for normal digestion. Definitions of lactose maldigestion and lactose intolerance were distinguished. The 9 essential nutrients (**9EN**) EM was designed to educate panelists about the benefits of consuming dairy products and what vitamin and mineral deficiencies may occur with dairy avoidance. It was explained that dairy products are a good to excellent source of 9 essential nutrients that are important to human health. The prebiotics and probiotics EM was designed to educate panelists about the terms probiotic (health-promoting bacteria) and prebiotic (food for bacteria). It provided definitions and examples of each and showed how lactose can act as a prebiotic. A key point was that even lactose maldigesters can benefit from small amounts of lactose because it can provide nutrition to healthy bacteria in the gut.

Panelists were then asked to take a 21-question Qualtrics post-survey (running time approximately 42–48 min), which asked about knowledge gained during the session (5 questions), purchase intent (12), and questions to test retention of lesson material (4). Panelists were given $15 cash and asked to sign a receipt (running time approximately 55–62 min).

One month following each NFG, survey links were sent to NFG participants. The survey had 21 questions about behaviors toward food packaging (2 questions), questions to test retention of lesson material (4), purchasing and consumption behaviors since the NFG (11), and purchase intent for potential new dairy products (4).

Data were downloaded from Qualtrics into Excel (Microsoft Corp., Redmond, WA). To measure the effectiveness of EM on dairy purchasing and consumption, categorical response options (e.g., a gallon of milk) were converted into quantitative answers, based upon the recommended servings of dairy foods (USFDA, 2022). Standardized serving sizes included 1.5 oz. cheese, 3.35 oz. ice cream, 8 fluid oz. milk, and 6 oz. yogurt. Distribution analyses of demographics, purchasing, and consumption data were conducted in JMP (version 15, SAS Institute Inc., Cary, NC). To evaluate the effectiveness of EM on learning by individuals, responses to quiz questions were coded as incorrect (0) or correct (1), then the nonparametric Wilcoxon signed-rank test was conducted in JMP to assess if knowledge was acquired between the NFG pre-survey and post-survey, if it was retained between the post-survey and 1-mo follow-up survey, and to determine if purchasing or consumption differed between before and after participating in NFG. An α-level of 0.05 was selected for determination of significance.

A total of 4,542 adults completed the screening survey (2,131 in IA and 2,411 in KS). After the removal of repeats, incomplete data, and funneling to find IDC, 566 people in Iowa and 421 in Kansas were eligible for and were invited to participate in NFG. Thus, 22% of the initially surveyed population (1) were located within driving distance, (2) completed the survey (spent adequate time on it), (3) consumed less than 3 servings of dairy per day, (4) did not have a diagnosed dairy intolerance, and (5) indicated an interest in participating in a focus group. A total of 195 (94 in IA and 101 in KS) participated in the NFG; 30% identified themselves as male and 70% as female. College-age participants predominated, with 52% between 18 and 24 yr of age, 17% between 25 and 34, 13% between 35 and 44, and 18% aged 45 and over. White participants predominated (78%), followed by 9% Asian American or Asian origin; 7% Latin American, Latin, or Spanish origin; 1% African American or African origin; and 3% of “other” or “prefer not to answer.” Lactose intolerance is more prevalent in people of African American, African, Asian American, Asian, Latin American, Hispanic, Latino, and Spanish origin than people of northern European (White/Caucasian) origin (Fassio et al., 2018), which may partially explain the lack of more diverse panelists.

Since dairy products contribute a good to excellent source of essential nutrients, it was considered important to teach participants how to interpret %DV labeling. To evaluate learning, panelists were asked “True or false: A Daily Value (%DV) of 5% or less of a nutrient per serving is low, and 20% DV or more of a nutrient per serving is high,” 3 times (pre-survey, post-survey, and follow-up survey). In the pre-survey, 86% were able to respond correctly (true), showing either a good initial understanding of the concept, or good guessing (Table 1). Nonetheless, a significant number of participants learned the information, as demonstrated by the 96.4% correct response rate on the post-survey (*P* < 0.05). One month after the NFG, the majority of participants (91.5%) correctly answered the question. However, this was a significant (*P* < 0.05) decrease from the NFG post-survey, so the information was not retained by all who learned it. Thus, some improvements may be necessary for future interventions to increase learning, test knowledge, and increase retention. For instance, providing additional information about using %DV to make nutritional decisions, later in the lesson, might help cement the knowledge.

**Table 1:** Percent incorrect and correct responses to quiz questions asked immediately before (Pre-), after (Post-), and 1 mo after (follow-up) nominal focus group sessions, by treatment group

Quiz topic	Pre- (n = 195)^1^				Post- (n = 195)^1^				Follow-up (n = 169)^1^			
	Control (n = 50)	Lactose maldigestion (n = 42)	9 essential nutrients (n = 53)	Prebiotics and probiotics (n = 49)	Control (n = 50)	Lactose maldigestion (n = 42)	9 essential nutrients (n = 53)	Prebiotics and probiotics (n = 49)	Control (n = 43)	Lactose maldigestion (n = 32)	9 essential nutrients (n = 47)	Prebiotics and probiotics (n = 49)
Daily value												
% Incorrect	10	10	12	24	0	2	8	4	5	9	7	14
% Correct	90	90	88	76	100	98	92	96	95	91	93	86
Average % correct	86.0^B^				96.4^A^				91.5^B^			
Lactose calculation												
% Incorrect					8	5	15	21	23	24	27	28
% Correct	NA^2^	NA	NA	NA	92	95	85	79	77	76	73	72
Average % correct	NA				87.1^A^				74.1^B^			
9 essential nutrients												
% Incorrect	88	96	90	93	94^b^	100^b^	8^a^	90^b^	86^b^	94^b^	38^a^	77^b^
% Correct	13	4	10	7	6	0	92	10	14	6	63	23
Average % correct	8.4^B^				35.3^A^				28.8^A^			

A,B Percent correct values not sharing the same superscript within a row significantly differ (Wilcoxon signed-rank test, *P* < 0.05).

a,b Percent incorrect values not sharing the same superscript within a row and survey type significantly differ (Wilcoxon signed-rank test, *P* < 0.05).

1 Missing values (question skipped) are not included in mean correctness scores.

2 NA = question not asked in the specified survey.

Participants were taught how to read NFP and were given specific guidance on how to determine the amount of lactose in a food product based on an NFP that was provided to them. Specifically, panelists were shown a commercial yogurt NFP and asked, “Using the Nutrition Facts label of this low-fat vanilla yogurt, how much lactose is in a serving?” The question was complex because it required them to know where to look on the NFP and involved arithmetic. They were taught that, because total sugars were 14 g and added sugars were 8 g, the correct answer in the multiple-choice question should be 6 g of lactose. Panelists were not asked this question in the pre-survey. Nearly all participants got the correct answer (87.1%) at the end of the NFG (post-survey; Table 1). After 1 mo, the correctness dropped to 74% (*P* < 0.05). Only 13% of NFG participants were unable to retain the information that was taught to them, suggesting the effectiveness of the lesson. For future interventions, including a second example (e.g., with milk, cheese, or ice cream) may improve learning and help cement the information in the minds of participants.

Although only one treatment group received the 9EN EM, all participants were asked, 3 times, the question, “How many essential nutrients are naturally found in dairy products (milk, yogurt, cheese, ice cream)?” Multiple-choice options included the following: I don't know, none, 1, 3, 5, 7, or 9. Only 8.4% of panelists got the correct answer in the NFG pre-survey, and there were no differences in mean correctness scores among the 4 treatment group populations (Table 1). This was expected because no one had received the EM yet, demonstrating that participants had little to no prior knowledge on the topic. After the NFG, those who received the 9EN EM got the question right more often (92%) than those who did not (*P* < 0.05), showing that the EM was effective (Table 1). After a month, this trend continued. The 9EN NFG participants' correctness was 63%—significantly (*P* < 0.05) higher than the other 3 treatment groups. Differences between groups were less pronounced than immediately after the NFG lesson, demonstrating that some of the individuals who received the lesson were not able to retain the information after 1 mo. Additionally, several may have gotten the answer correct by searching for it on the internet while taking the follow-up survey, which may explain why the overall correctness (28.8%) was significantly (*P* < 0.05) higher than on the pre-survey.

Pre-survey data validated that the target audience, IDC, were effectively screened to participate in the NFG. On average, participants purchased only 3.48 servings of dairy products (cheese, ice cream, milk, and yogurt) per week and consumed only 5.95 servings per week (Table 2). Because this is less than the recommended 21 servings per week, these IDC may not have been consuming enough calcium, riboflavin, and vitamin D (USDA and USDHHS, 2020).

**Table 2:** Mean servings of dairy products purchased and consumed by those who usually consume less than 3 servings of dairy per day in Iowa and Kansas, calculated from responses in the pre-survey and 1-mo follow-up survey

Item	Mean pre-survey servings	Mean follow-up survey servings	% change in servings	*P*-value
Cheese purchased	0.96	1.22	27.08	0.006
Ice cream purchased	0.74	0.89	20.27	0.05
Milk purchased	1.47	1.87	27.21	0.002
Yogurt purchased	0.32	0.40	25.00	0.04
Total servings purchased	3.48	4.38	25.86	
Cheese consumed	1.79	2.20	22.91	<0.0001
Ice cream consumed	1.27	1.52	19.69	0.008
Milk consumed	2.27	3.47	52.86	<0.0001
Yogurt consumed	0.65	0.82	26.15	0.02
Total servings consumed	5.95	8.01	34.68	

Attending NFG had a positive effect on dairy product purchasing, with a 27% increase for cheese, 20% increase for ice cream, 27% increase for milk, and 25% increase for yogurt between the NFG pre-survey and 1-mo follow-up survey (*P* ≤ 0.05, Table 2). Average dairy product purchasing increased to 4.38 servings per week, a 26% increase. Average consumption of each dairy product also increased after attending NFG, with a 23% increase in cheese, 20% increase in ice cream, 53% increase in milk, and 26% increase in yogurt consumption (*P* < 0.05, Table 2). Total dairy consumption increased to 8.01 servings per week, a 35% increase.

When the behavior of individuals was evaluated by conducting the Wilcoxon signed-rank test, additional differences were revealed (Table 3). Purchasing of dairy products increased on average by 0.20 servings per person/week, and consumption increased 0.52 servings per person/week. Similar trends were seen for consumption. Although significant increases were seen for all products, increases in consumption were less than half a serving per week for cheese, ice cream and yogurt. The exception was milk. The average individual consumption increase was 1.26 servings per week; consumption increased by at least 1 serving per week in every treatment group. For both purchasing and consumption, increases in total dairy products purchased or consumed were significant (*P* < 0.05) in all treatment groups.

**Table 3:** Changes in servings of dairy products purchased and consumed by individuals who usually consume less than 3 servings of dairy per day (Wilcoxon signed-rank test) in Iowa and Kansas, separated by educational message

Item	Control (n = 43)		Lactose maldigestion (n = 30)		9 essential nutrients (n = 49)		Prebiotics and probiotics (n = 43)		Full dataset (n = 165)	
	Mean difference	Wilcoxon *P*-value	Mean difference	Wilcoxon *P*-value	Mean difference	Wilcoxon *P*-value	Mean difference	Wilcoxon *P*-value	Mean difference	Wilcoxon *P*-value
Cheese purchasing	0.11	0.1	0.25	*	0.14	0.05	0.34	0.06	0.21	**
Ice cream purchasing	0.11	0.2	0.003	0.5	0.12	0.09	0.25	0.005	0.25	**
Milk purchasing	0.35	***	0.39	0.08	0.30	*	0.33	**	0.32	***
Yogurt purchasing	0.08	0.09	−0.001	0.5	0.13	**	0.08	0.07	0.08	**
All dairy product purchasing	0.20	***	0.17	*	0.19	***	0.26	***	0.20	***
Cheese consumption	0.16	0.2	0.75	***	0.26	0.05	0.48	***	0.39	***
Ice cream consumption	0.07	0.3	0.22	0.11	0.43	**	0.29	*	0.26	**
Milk consumption	1.35	***	1.89	**	1.00	***	1.51	***	1.26	***
Yogurt consumption	0.17	*	0.13	0.2	0.31	***	0.17	0.2	0.20	**
All dairy product consumption	0.42	***	0.56	***	0.48	***	0.62	***	0.52	***

* *P* < 0.05,

** *P* < 0.01,

*** *P* < 0.001.

Taken together, these results suggest that participation in dairy nutrition educational session can positively affect purchasing and consumption of dairy products. Nonetheless, because participants did not increase consumption to the recommended 21 servings/week, nutritional benefits may not be apparent unless dairy consumption continues long term or additional increases are made. Improvements in the materials and delivery could potentially enhance the effect of the lessons and lead to meaningful behavior change. Intentional, repeated positive messaging about dairy products nutrition are encouraged to yield meaningful increases in dairy purchasing and consumption.

Correct responses to surveys validated that IDC who participated in NFG learned about the meaning of daily value, how to calculate lactose from a NFP, and essential nutrients in dairy foods. Participation in NFG significantly (*P* < 0.05) increased dairy purchasing and consumption, but increases were not high enough for the IDC to meet the recommended intake of 21 servings per week. Overall, this study demonstrates that carefully constructed dairy nutrition concepts can be learned and retained by consumers. However, it also revealed the complexity of trying to motivate the retention of knowledge and meaningful increases in dairy purchasing and consumption. The materials and methodology in this study can be relatively easily revised, by improving the clarity of lessons and survey questions, to not only improve learning about dairy product nutrition, but to promote lasting change in positive behaviors with dairy products.
